# 

**Published:** 1897-02-13

**Authors:** 


					THE HOSPITAL. ABSOLUTELY PURE, therefore BEST, Fhb. 13th, 1897,
t/?ADof.4?YS FfU b CAD.BURY'S
COCOA . FIT Ini JK? COOOA
S^*4J?Jrp",,",p'ese" % m ?3s?> NO alkalies used.
soorr^ Western Infirm art * ~ 1 - ^ * NQRLQLKmi^norwich hospisaj.
No. 542> ivol. XXI.
Saturday,
Feb. 13th, 1897.
PRICE TWOPENCE.
[Registered at the General Post Office as a Newspaper for
[Be&?nb.io? m th. United Ei.gdom and Abroad.] SuSSt KSsSi S5S

				

